# Chelator-Accelerated One-Pot ‘Click’ Labeling of Small Molecule Tracers with 2-[^18^F]Fluoroethyl Azide

**DOI:** 10.3390/molecules18055335

**Published:** 2013-05-10

**Authors:** Eva Galante, Bent Wilhelm Schoultz, Matthias Koepp, Erik Årstad

**Affiliations:** 1 Department of Chemistry and Institute of Nuclear Medicine, University College London, 235 Euston Road (T-5), London, NW1 2BU, UK; E-Mail: e.galante@ucl.ac.uk; 2 Department of Chemistry, University of Oslo, PO Box 1033, Blindern N-0315, Oslo, Norway; E-Mail: b.w.schoultz@kjemi.uio.no; 3 Department of Clinical and Experimental Epilepsy, Institute of Neurology, University College London, 33 Queen Square, London, WC1N 3BG, UK; E-Mail: m.koepp@ucl.ac.uk

**Keywords:** fluorine-18, click chemistry, BPDS, 2-[^18^F]fluoroethyl azide, 6-halopurine, automated synthesis, 1,2,3-triazole, PET, radiotracer

## Abstract

2-[^18^F]Fluoroethyl azide ([^18^F]FEA) can readily be obtained by nucleophilic substitution of 2-azidoethyl-4-toluenesulfonate with [^18^F]fluoride (half-life 110 min), and has become widely used as a reagent for ‘click’ labeling of PET tracers. However, distillation of [^18^F]FEA is typically required, which is time-consuming and unpractical for routine applications. In addition, copper(I)-catalyzed cycloaddition of [^18^F]FEA with non-activated alkynes, and with substrates containing labile functional groups, can be challenging. Herein, we report a highly efficient and practical ligand-accelerated one-pot/two-step method for ‘click’ labeling of small molecule tracers with [^18^F]FEA. The method exploits the ability of the copper(I) ligand bathophenanthrolinedisulfonate to accelerate the rate of the cycloaddition reaction. As a result, alkynes can be added directly to the crude reaction mixture containing [^18^F]FEA, and as cyclisation occurs almost immediately at room temperature, the reaction is tolerant to labile functional groups. The method was demonstrated by reacting [^18^F]FEA with a series of alkyne-functionalized 6-halopurines to give the corresponding triazoles in 55–76% analytical radiochemical yield.

## 1. Introduction

Labeling with ^18^F is challenging due to the time constraints imposed by the short half-life (110 min), the need to manipulate minute amounts of [^18^F]fluoride, and the need for remote automated synthesis of tracers for clinical use. [^18^F]Fluoride can readily be produced with high specific activity (SA), and is by far the most common source of ^18^F for tracer synthesis. Labeling is typically achieved by nucleophilic aliphatic substitution of suitable leaving groups, such as sulfonates, with [^18^F]fluoride. Yet, ^18^F-labeling of aliphatic groups is sensitive to the presence of hydrogen bond donors, as well as neighbouring group effects, and as a result tracer synthesis often requires the use of prosthetic labeling reagents. The copper(I)-catalyzed cycloaddition reaction of azides and alkynes to give 1,2,3-triazoles has recently emerged as a highly versatile method for conjugation of ^18^F-labeled ‘click’ reagents to small molecules and peptides [1,2,3,4]. Despite the broad substrate scope, the efficiency of the reaction is dependent on the nature of the ‘click’ partners, and conjugation to substrates with low reactivity or labile functional groups can be challenging [5]. In addition, purification of the intermediate labeling reagent is often required prior to the cycloaddition reaction in order to limit the formation of side products. 

**Figure 1:**
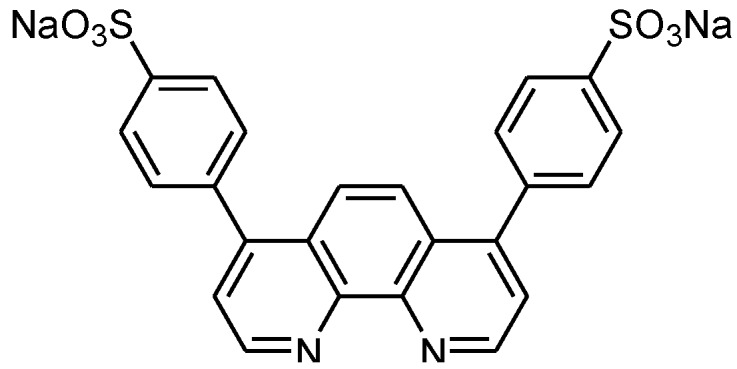
Chemical structure of BPDS.

Among the array of ‘click’ reagents reported, 2-[^18^F]fluoroethyl azide ([^18^F]FEA) is attractive as its small size makes it particularly suited for labeling of small molecule tracers [2,5,6,7,8]. However, purification can only be achieved by distillation, which is delicate, poorly compatible with automated synthesis modules, and also potentially hazardous as it can result in release of gaseous [^18^F]FEA. Herein, we reported a convenient and highly efficient one-pot method for ‘click’ labeling with [^18^F]FEA. The method exploits the ability of the copper chelator bathophenanthrolinedisulfonate (BPDS, Figure 1) to accelerate the cycloaddition reaction, which overcomes the need to purify the intermediate [^18^F]FEA, and enables conjugation to precursors with labile functional groups. The method was demonstrated by reacting [^18^F]FEA in the form of a crude reaction mixture with a series of alkyne-functionalized 6-halopurines in the presence of copper(II)/ascorbate and BPDS to give the corresponding triazoles in 40–57% isolated radiochemical yield and with >99% radiochemical purity. 

## 2. Results and Discussion

### 2.1. Chemistry

**Scheme 1:**
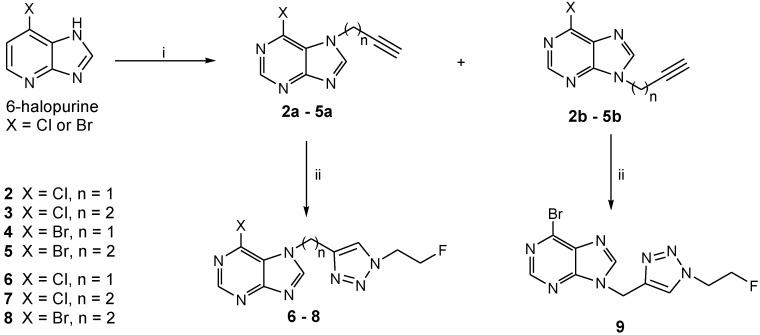
Synthesis of the 6-halopurine alkynes **2**–**5** and the corresponding triazoles **6**–**9**.

The alkyne-functionalized 6-halopurines **2**–**5** (Scheme 1) and the corresponding triazole derivatives **6**–**9** were synthesized as labeling precursors and non-radioactive fluorinated reference compounds, respectively. The alkyne group was initially introduced into the purine scaffold by a base-promoted alkylation of 6-bromopurine and 6-chloropurine using propargyl bromide or *p*-tosylate-1-butyne (**1**) as previously reported [9]. The reaction provided both the N7-alkynyl (**2a**–**5a**, 10–20%) and the N9-alkynyl 6-halopurines (**2b**–**5b**, 40–60%), which were separated by flash chromatography. The N7-isomers were distinguished from the corresponding N9-isomers by down field shifts of H-2 and H-8 protons in ^1^H-NMR [10,11]. Compounds **2a**, **3a**, **4b** and **5a** were subsequently treated with FEA in the presence of catalytic amount of copper(II) sulfate and sodium ascorbate to provide the corresponding triazoles **6**–**9** in moderate to good yields.

### 2.2. Radiochemistry

**Figure 2:**
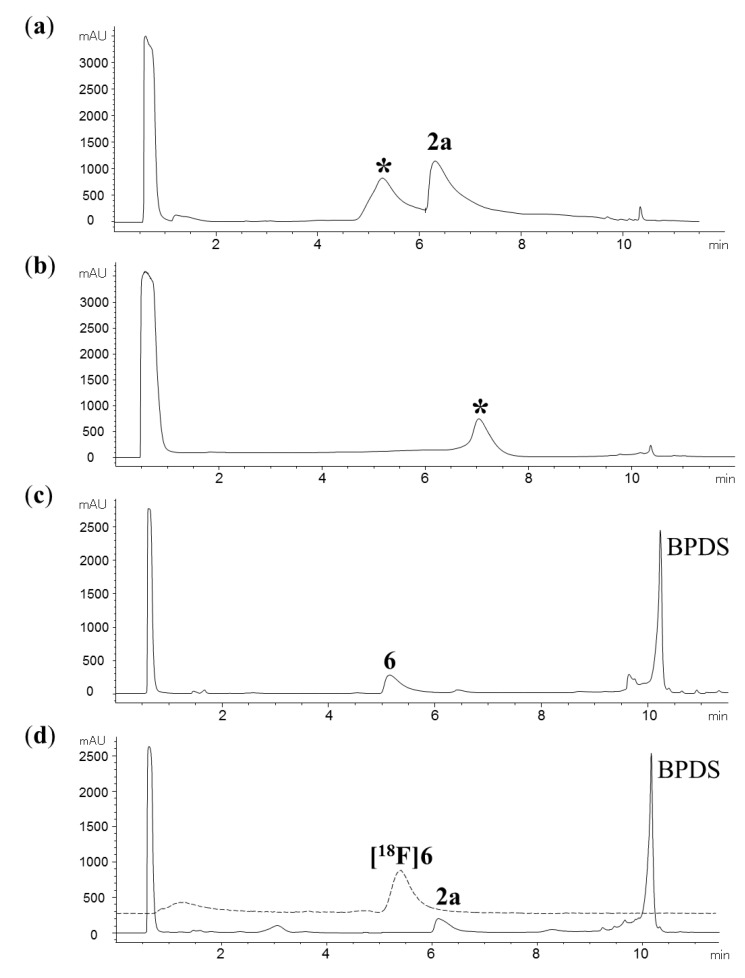
Analytical HPLC profiles (UV at 265 nm) of the reaction mixtures resulting from treatment of **2a** with non-radioactive FEA (1 equiv.) in the presence of excess copper(II) sulfate and (**a**) sodium ascorbate or (**b**) ascorbic acid. Unknown side products are labeled with *; (**c**) Reaction of **2a** and FEA in the presence of the BPDS-copper(I) catalytic system; (**d**) Addition of **2a**, copper(II) sulfate, BPDS and ascorbate to a crude reaction mixture containing [^18^F]FEA resulted in clean formation of the triazole [^18^F]**6** (radioactivity profile shown as dotted line).

As formation of the N7-alkylated triazoles **6**–**8** under preparative conditions gave moderate yields (32–40%) with apparent decomposition of the corresponding precursors, we initially used non-radioactive FEA and the alkyne **2a** to optimize the conditions for the cycloaddition reaction. Attempts to increase the reaction rate by the use of excess copper(II) sulfate and sodium ascorbate relative to the alkyne **2a** failed to give the triazole **6** (15 min, rt), and instead resulted in formation of an unknown side product with a similar HPLC retention time to that of the target compound **6** [Figure 2(a)]. The use of ascorbic acid to allow formation of copper(I) under acidic conditions was also unsuccessful, and resulted in complete consumption of the precursor **2a** with formation of another unidentified side product [Figure 2(b)]. To exclude the possibility that side product formation was caused by the *in situ* reduction of copper(II) sulfate, we investigated copper(I) chloride and triethylamine as an alternative catalytic system. However, we were unable to obtain the target triazole **6**, and instead obtained product mixtures with similar HPLC profiles to that observed for the reaction of **2a** and FEA in the presence of copper(II)/ascorbate [Figure 2(a)]. 

Purines are known to form complexes with copper ions [12,13], and it is plausible that the combined interactions of the alkyne group and nitrogens in **2a** with copper(I) results in formation of a complex that impairs subsequent cyclisation with FEA through unfavorable steric or electronic interactions. Instead of increasing the rate of the cycloaddition reaction, addition of excess copper(I) relative to the alkyne **2a** may therefore favor hydrolysis of the precursor **2a** by activation of the chlorine in the 6-position. To explore this possibility, we investigated the use of BPDS as an auxiliary copper(I) chelator. The BPDS-copper(I) catalytic system was first identified from a fluorescent assay [14], and has since been used to accelerate triazole formation for reactions with peptides and substrates with high molecular weight for which the copper(II)/ascorbate system alone often provides unsatisfactory results [15,16]. Remarkably, in the presence of BPDS, copper(II) sulfate and sodium ascorbate, the cycloaddition of **2a** with FEA occurred almost immediately at room temperature to give the target triazole **6** [Figure 2(c)].

Encouraged by the superb efficiency of the cycloaddition reaction in the presence of the BPDS-copper(I) catalytic system, we explored the possibility of preparing the triazoles [^18^F]**6**–[^18^F]**9** in an one-pot reaction without distillation of the intermediate [^18^F]FEA. Indeed, sequential addition of alkyne **2a**, copper(II) sulfate, BPDS and sodium ascorbate to a crude reaction mixture containing [^18^F]FEA resulted in formation of [^18^F]**6** in 60% analytical radiochemical yield (RCY) within 1 min at room temperature. Provided that the alkyne **2a** was used in excess relative to copper(II) sulfate and BPDS, no other radioactive product was observed, and the precursor **2a** remained largely intact after the reaction [Figure 2(d)]. The apparent absence of non-radioactive triazoles in the product mixture was unexpected and suggests that the cycloaddition rate of FEA to alkynes far exceeds that for the corresponding 2-azidoethyl-4-toluenesulfonate precursor under these conditions. Comparable results were obtained for formation of triazoles [^18^F]**7**–**9**, which were obtained in 55–76% analytical and 40–57% isolated (decay-corrected) radiochemical yields (Table 1). 

**Table 1:** Radiochemical yields for formation of [^18^F]**6**–[^18^F]**9**.

Entry	Product		RCY a (%)
1	[^18^F]**6**	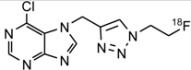	60 ± 1 (41) ^b^
2	[^18^F]**7**	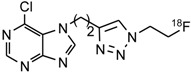	76 ± 1 (40) ^b,c^
3	[^18^F]**8**	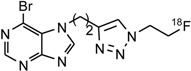	55 ± 2 (55) ^b^
4	[^18^F]**9**	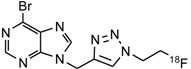	76 ± 1 (57) ^b^

^a^ Average analytical decay-corrected radiochemical yields ± standard deviation (n = 3). ^b^ Isolated RCY in brackets (n = 1). ^c^ Isolated yield obtained from a fully automated radiosynthesis.

### 2.3. Automated radiosynthesis of purine [^18^F] *7*

**Figure 3:**
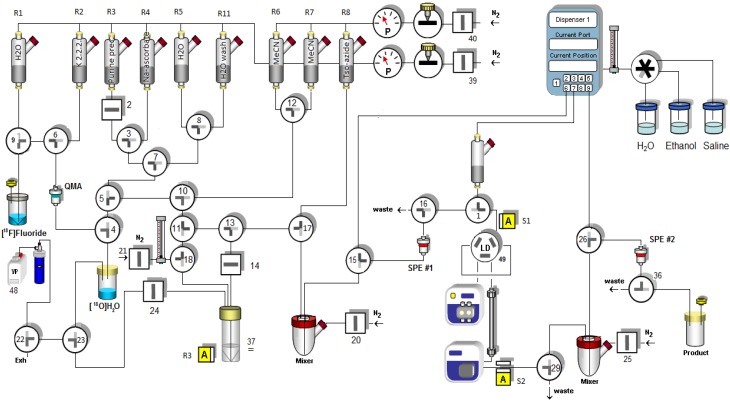
Schematic representation of the configuration of the HBIII synthesis module.

To evaluate the suitability of the one-pot method for routine tracer production we established an automated synthesis of the triazole-functionalized purine [^18^F]**7** using a HBIII module (Scintomics). The configuration of the synthesis module is illustrated in Figure 3. [^18^F]Fluoride in water (~1.5 GBq) was trapped on an ion-exchange cartridge (QMA cartridge Sep-Pak® light, Waters) and released with a solution of Kryptofix 222 and potassium carbonate (0.5 mL of a solution 30 mM: 15 mM in acetonitrile:water = 85:15). Following azeotropic drying with anhydrous acetonitrile at 90 °C (2 × 0.5 mL), a solution of 2-azidoethyl-4-toluenesulfonate (8.4 µmol) in acetonitrile (0.4 mL) was added and the resulting mixture was allowed to react for 15 min at 80 °C, which resulted in formation of [^18^F]FEA in 80–90% analytical RCY. The reactor vial was cooled to room temperature with a stream of nitrogen, and a solution containing the alkyne **3** (10 µmol), CuSO_4_·5H_2_O (8 µmol) and BPDS (8 µmol) in a mixture of acetonitrile (0.15 mL) and phosphate buffer (0.2 mL, 0.1 M, pH = 7.4) was added, followed by a solution of sodium ascorbate (40 µmol in 0.1 mL H_2_O). After 1 min at room temperature the reaction was diluted with H_2_O (1 mL). HPLC analysis showed complete conversion of [^18^F]FEA to give [^18^F]**7** in 75% analytical RCY. To enable purification on a semi-preparative column, we investigated the use of cartridge-based methods to clean up the product mixture prior to HPLC injection. However, the high polarity of [^18^F]**7** made cartridge trapping challenging, and also complicated subsequent purification by HPLC. 

**Figure 4:**
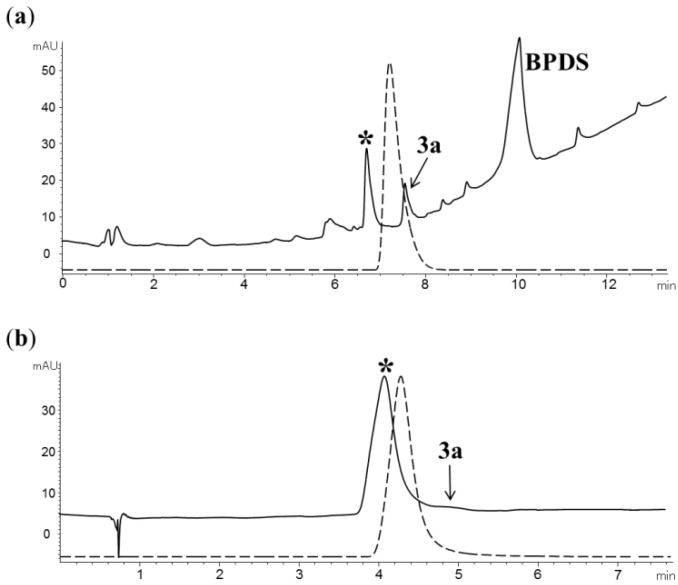
Analytical radio-HPLC profile (dotted line) and UV profile (265 nm, solid line) of [^18^F]**7** obtained from the automated one-pot synthesis after using (**a**) Sep-Pak® Plus (Luna C18(2) column) and (**b**) and C-18 Sep-Pak® Light cartridge (Chromolith® performance column, RP18-e). Unidentified side product is labeled with *.

In order to maximize the trapping efficiency of [^18^F]**7**, we evaluated a number of solid phases, sizes of cartridges, and solvent systems. The highest RCY was obtained by dilution of the product mixture with water (30 mL) to give a total of 2% acetonitrile, followed by trapping of the crude product on a C-18 cartridge (Sep-Pak® Plus, Waters). The product was eluted with 25% acetonitrile in water (2 mL), the resulting solution was diluted with water (3 mL) and subsequently purified by HPLC (Chromolith® RP18-e using 9% acetonitrile with 0.1% formic acid as the eluent) to give [^18^F]**7** in 40% decay-corrected RCY. While the radiochemical purity was > 99% [Figure 4(a)], the specific activity was still below 1 GBq/μmol. The use of a smaller cartridge (C-18 Sep-Pak® light, Waters) allowed release of the product in a lower solvent volume (20% ethanol in water, 1 mL), however the trapping was less efficient and led to loss of 15–20% of the product. The resulting solution was diluted with water (1 mL) and purified by HPLC as described above. The collected fraction was diluted with water to give a total concentration of 5% in acetonitrile, the product was trapped on C-18 Sep-Pak® light cartridge (Waters), the cartridge was washed with water (1 mL), and the product eluted with 20% ethanol in saline (0.8 mL). The resulting solution was diluted with saline for injection (2.4 mL) to give a total of 5% ethanol, and filtered on sterile filter (Millex-GV 0.22 µm, 13 mm, Millipore). When starting with 1–1.5 GBq [^18^F]fluoride, the formulated product [^18^F]**7** was obtained in 9 ± 2% (n = 3) decay-corrected RCY after sterile filtration, with >99% radiochemical purity and a specific activity in the range of 5–7 GBq/μmol [Figure 4(b)]. The total synthesis time was 105 min.

An inherent limitation of the one-pot method for cycloaddition of [^18^F]FEA with alkynes is that the total contents of the product mixture exceed the typical capacity of semi-preparative HPLC columns, and hence cartridge-based purification is required prior to sample loading. The high polarity of [^18^F]**7** presented particular challenges in this respect as cartridge trapping and HPLC purification only was achievable when using aqueous solutions with very low concentrations of organic solvents. The poor retention of [^18^F]**7** on reverse-phase stationary phases necessitated extensive dilution of product solutions, which added to the overall synthesis time, and also made it difficult to obtain the target compound with high specific activity. However, such complications should not affect tracers with more favorable physiochemical properties, in which case a shorter synthesis time with improved RCY and higher specific activity is likely to be achieved. While the outcome of the cycloaddition reaction will depend on the nature of the alkyne precursor, and the conditions used, in our hands we observed no competing reactions that are likely to complicate tracer purification. 

It should be noted that one-pot ‘click’ labeling with [^18^F]FEA have been reported previously [17], and that BPDS has been used for one-pot labeling with other ‘click’ reagents [16]. However, in the absence of a chelator to accelerate the reaction rate, one-pot labeling with [^18^F]FEA is limited to highly reactive alkyne precursors. For ‘click’ reagents other than [^18^F]FEA, which readily can be purified with solid-phase extraction, one-pot ^18^F-labeling offer fewer benefits, which may explain the limited use of BPDS despite the astonishing improvement in reaction rates that can be achieved with this chelator. 

## 3. Experimental

### 3.1. General

All reagents were purchased from Sigma-Aldrich (Dorset, UK) and used without further purification. NMR spectra were recorded on Bruker Avance (500 MHz or 600 MHz) spectrometers operating at the frequency of 500 or 600 MHz for ^1^H, and 125 or 150 MHz for ^13^C. Chemical shifts (δ) are reported in ppm downfield from the internal standard tetramethylsilane. High resolution mass spectra were recorded on a thermo Finnigan MAT900xp (CI/EI) or a Waters LCT Premier XE (ES) mass spectrometers. No-carrier-added aqueous [^18^F]fluoride was provided by St Thomas’ Hospital, King’s College London, UK. HPLC analyses were performed with an Agilent 1200 series system equipped with a diode array UV detector (results described are for UV at 265 nm), and a Raytest GABI star NaI detector. Analytical runs and HPLC purifications were carried out on a Chromolith® performance column, RP18-e, 100 × 4.6 mm and 100 × 10 mm (Merck), respectively. The solvent systems used were water and methanol, both containing 0.1% of formic acid. The flow rate was 3 mL/min for analytical and 5 mL/min for semi-preparative columns, respectively. Automated synthesis was carried out on HBIII module (Scintomics, Lindach, Germany). Isolated radiochemical yields (RCY) were measured using a Curiementor^®^ 4 ion-chamber (PTW).

### 3.2. Chemistry

#### 3.2.1. General Procedure for Preparation of the Alkyne-functionalized 6-Halopurines

To a mixture of the 6-halopurine (1.0 equiv.) and NaH (60% dispersed in oil, 1.1 equiv.) in anhydrous DMF was added the alkylating agent (1.3 equiv.) under inert atmosphere. After stirring for 24 h at room temperature, the reaction mixture was diluted with dichloromethane (DCM) and extracted three times with water. The organic layer was dried over MgSO_4_ and the solvent was removed under reduced pressure. The residue was purified by flash chromatography on silica gel using a gradient of DCM and ethyl acetate (from 0% to 40% of ethyl acetate) to give the N7- and the N9-alkynyl analogues. 

*6-Chloro-7-propargylpurine* (**2a**)* and 6-chloro-9-propargylpurine* (**2b**). A mixture of 6-chloropurine (500 mg, 3.24 mmol), and NaH (60% dispersed in oil, 142 mg, 3.56 mmol) in anhydrous DMF (15 mL) was treated with propargyl bromide (468 µL, 4.21 mmol) as described under the general alkylation procedure to give **2a** (105 mg, 17%) and **2b** (363 mg, 58%). Compound **2a**: ^1^H-NMR (CDCl_3_, 600 MHz): δ 8.89 (s, 1H, H-2), 8.49 (s, 1H, H-8), 5.29 (d, *J* = 2.58 Hz, 2H, CH_2_C), 2.68 (t, *J* = 2.58 Hz, 1H, CCH). ^13^C-NMR (CDCl_3_): δ 162.2, 153.0, 148.3, 143.3, 122.3, 77.4, 75.1, 37.4. Accurate mass (EI-MS) *m/z* calcd for C_8_H_5_ClN_4_ (M)^+^ 192.0197, found 192.0204. Mp: 102 ± 1 °C. Compound **2b**: ^1^H-NMR (CDCl_3_, 500 MHz): δ 8.74 (s, 1H, H-2), 8.33 (s, 1H, H-8), 5.06 (t, *J* = 2.46 Hz, 2H, CH_2_C), 2.58 (t, *J* = 2.55 Hz, 1H, CCH). ^13^C-NMR (CDCl_3_): δ 152.3, 151.3, 144.4, 131.6, 76.0, 75.1, 33.8. Accurate mass (EI-MS) *m/z* calcd for C_8_H_5_ClN_4_ (M)^+^ 192.0197, found 192.0190. Mp: 120 ± 1 °C.

*6-Chloro-7-(1-butyne)purine* (**3a**)* and 6-chloro-9-(1-butyne)purine* (**3b**). A mixture of 6-chloropurine (500 mg, 3.24 mmol), and NaH (60% dispersed in oil, 142 mg, 3.56 mmol) in anhydrous DMF (15 mL) was treated with 4-tosyloxy-1-butyne (1.56 g, 4.21 mmol) as described under the general alkylation procedure to give **3a** (109 mg, 19%) and **3b** (466 mg, 41%). Compound **3a**: ^1^H-NMR (CDCl_3_, 600 MHz): δ 8.89 (s, 1H, H-2), 8.34 (s, 1H, H-8), 4.64 (t, *J* = 5.25 Hz, 2H, C*H_2_*CH_2_C), 2.84 (dt, *J* = 5.25 Hz, *J* = 2.15 Hz, 2H, CH_2_C), 2.01 (t, *J* = 2.15 Hz, 1H, CCH). ^13^C-NMR (CDCl_3_): δ 162.4, 152.7, 149.7, 142.8, 122.2, 78.7, 73.0, 45.9, 21.9. Accurate mass (EI-MS) *m/z* calcd for C_9_H_7_N_4_Cl (M)^+^ 206.0353, found 206.0359. Mp: 79 ± 1 °C. Compound **3b**: ^1^H-NMR (CDCl_3_, 500 MHz): δ 8.74 (s, 1H, H-2), 8.25 (s, 1H, H-8), 4.46 (t, *J* = 5.30 Hz, 2H, C*H_2_*CH_2_C), 2.83 (m, 2H, CH_2_C), 2.06 (t, *J* = 2.15 Hz, 1H, CCH). ^13^C-NMR (CDCl_3_): δ 152.1, 151.7, 151.3, 145.6, 131.8, 79.4, 72.4, 43.2, 20.1. Accurate mass (EI-MS) *m/z* calcd for C_9_H_7_N_4_Cl (M)^+^ 206.0353, found 206.0353. Mp: 77 ± 2 °C.

*6-Bromo-7-propargylpurine* (**4a**) * and 6-bromo-9-propargylpurine* (**4b**). A mixture of 6-bromopurine (500 mg, 2.51 mmol), and NaH (60% dispersed in oil, 111 mg, 2.76 mmol) in anhydrous DMF (15 mL) was treated with propargyl bromide (365 µL, 5.03 mmol) as described under the general alkylation procedure to give **4a** (115 mg, 19%) and **4b **(361 mg, 61%). Compound **4a**: ^1^H-NMR (CDCl_3_, 500 MHz): δ 8.81 (s, 1H, H-2), 8.51 (s, 1H, H-8), 5.33 (d, *J* = 2.56 Hz, 2H, CH_2_C), 2.67 (t, *J* = 2.56 Hz, 1H, CCH). ^13^C-NMR (CDCl_3_): δ 161.1, 152.8, 148.6, 133.3, 124.5, 77.4, 75.1, 37.2. Accurate mass (EI-MS) *m/z* calcd for C_8_H_5_BrN_4_ (M)^+^ 235.9697, found 235.9698. Mp: 103 ± 2 °C. Compound **4b**: ^1^H-NMR (CDCl_3_, 500 MHz): δ 8.73 (s, 1H, H-2), 8.36 (s, 1H, H-8), 5.06 (d, *J* = 2.60 Hz, 2H, CH_2_C), 2.57 (t, *J* = 2.60 Hz, 1H, CCH). ^13^C-NMR (CDCl_3_): δ 152.3, 150.1, 144.2, 143.4, 134.3, 76.0, 75.0, 33.8. Accurate mass (CI-MS) *m/z* calcd for C_8_H_6_BrN_4_ (M+H)^+^ 236.9775, found 236.9779. Mp: 154 ± 1 °C. 

*6-Bromo-7-(1-butyne)purine* (**5a**) * and 6-bromo-9-(1-butyne)purine* (**5b**). A mixture of 6-bromopurine (500 mg, 2.51 mmol), and NaH (60% dispersed in oil, 111 mg, 2.76 mmol) in anhydrous DMF (15 mL) was treated with 4-tosyloxy-1-butyne (732 mg, 3.27 mmol) as described under the general alkylation procedure to give **5a** (58 mg, 9%) and **5b **(276 mg, 44%). Compound **5a**: ^1^H-NMR (CDCl_3_, 600 MHz): δ 8.85 (s, 1H, H-2), 8.41 (s, 1H, H-8), 4.67 (t, *J* = 6.24 Hz, 2H, C*H_2_*CH_2_C), 2.85 (dt, *J* = 6.24 Hz, *J* = 2.34 Hz, 2H, CH_2_C), 2.10 (t, *J* = 2.64 Hz, 1H, CCH). ^13^C-NMR (CDCl_3_): δ 161.3, 152.7, 150.0, 133.1, 124.5, 78.7, 73.1, 45.6, 21.9. Accurate mass (EI-MS) *m/z* calcd for C_9_H_7_N_4_Br (M)^+^ 249.9848, found 249.9856. Mp: 97 ± 1 °C. Compound **5b**: ^1^H-NMR (CDCl_3_, 500 MHz): δ 8.67 (s, 1H, H-2), 8.25 (s, 1H, H-8), 4.44 (t, *J* = 5.0 Hz, 2H, C*H_2_*CH_2_C), 2.80 (m, 2H, CH_2_C), 2.05 (t, *J* = 2.59 Hz, 1H, CCH). ^13^C-NMR (CDCl_3_): δ 151.9, 150.4, 145.4, 143.3, 134.3, 79.4, 72.3, 43.2, 20.0. Accurate mass (EI-MS) *m/z* calcd for C_9_H_7_N_4_Br (M)^+^ 249.9848, found 249.9842. Mp: 94 ± 1 °C.

*2-Fluoroethyl azide* (**FEA**). To a solution of 2-fluoroethyl-4-toluenesulfonate [2] (1.1 g, 5.0 mmol) in dry DMF (15 mL) was added sodium azide (279 mg, 4.28 mmol). The resulting mixture was left to react for 24 h at ambient temperature, and used as a stock solution in subsequent reactions without any purification. *WARNING: Attempts to isolate neat 2-fluoroethyl azide may result in an explosion.*

#### 3.2.2. General Procedure for Cycloaddition of 2-fluoroethyl azide (FEA) with Alkyne-functionalized Purines

A solution of the alkyne-functionalized 6-halopurine (1.0 equiv.) in DMF was subsequently reacted with FEA (1.3 equiv.) in the presence of sodium ascorbate (0.1 equiv.) and CuSO_4_·5H_2_O (0.05 equiv.) at room temperature for 4 h. The reaction mixture was diluted with DCM and the organic phase was washed with water. The organic layer was dried with MgSO_4_ and the solvent was removed under reduced pressure. The residue was purified by flash chromatography on silica using a gradient of ethyl acetate and MeOH (from 0% to 10% of MeOH). 

*6-Chloro-7-[1-(2-fluoroethyl)-1H-*[1,2,3]*triazol-4-ylmethyl]-purine* (**6**). To a solution of **2a** (80 mg, 0.42 mmol) and FEA (48 mg, 0.54 mmol) in DMF (3 mL), was added sodium ascorbate (8.1 mg, 0.042 mmol) and CuSO_4_·5H_2_O (5.2 mg, 0.021 mmol) in H_2_O (0.5 mL) as described under the general procedure to give compound **6** in 40% yield (47 mg). ^1^H-NMR (CDCl_3_, 600 MHz): δ 8.87 (s, 1H, H-2), 8.49 (s, 1H, H-8), 7.75 (s, 1H, CH), 5.82 (s, 2H, CH_2_), 4.78 (dt, *J*_F,H_ = 46.69 Hz, *J*_1,2_ = 4.44 Hz, 2H, CH_2_F), 4.68 (dt, *J*_F,H_ = 27.13 Hz, *J*_1,2_ = 4.44 Hz, 2H, C*H*_2_CH_2_F). ^13^C-NMR (CDCl_3_): δ 162.1, 152.9, 149.4, 142.9, 142.2, 123.6, 122.2, 82.0, 80.9, 51.0, 50.9, 42.1. Accurate mass (CI-MS) *m/z* calcd for C_10_H_10_ClFN_7_ (M+H)^+^ 282.0670, found 282.0667. Mp: 143 ± 1 °C.

*6-Chloro-7-{2-[1-(2-fluoroethyl)-1H-*[1,2,3]*triazol-4-yl]-ethyl}-purine* (**7**). To a solution of **3a** (30 mg, 0.15 mmol) and FEA (17 mg, 0.19 mmol) in DMF (2 mL) was added sodium ascorbate (2.9 mg, 0.015 mmol) and CuSO_4_·5H_2_O (1.8 mg, 0.007 mmol) in H_2_O (0.2 mL) as described under the general procedure to give compound **7** in 32% yield (14 mg). ^1^H-NMR (CDCl_3_, 600 MHz): δ 8.87 (s, 1H, H-2), 8.34 (s, 1H, H-8), 7.27 (s, 1H, CH), 4.94 (t, *J*_1,2_ = 6.6 Hz, 2H, C*H_2_*CH_2_), 4.74 (dt, *J*_F,H_ = 46.8 Hz, *J*_1,2_ = 4.74 Hz, 2H, CH_2_F), 4.60 (dt, *J*_F,H_ = 27.1 Hz, *J*_1,2_ = 4.5 Hz, 2H, C*H*_2_CH_2_F), 3.34 (t, *J*_1,2_ = 5.50 Hz, 2H, CH_2_C*H_2_*). ^13^C-NMR (CDCl_3_): δ 162.2, 152.7, 149.8, 142.9, 142.7, 123.2, 122.2, 82.1, 81.0, 50.8, 50.6, 46.4, 28.0. Accurate mass (ESI-MS) *m/z* calcd for C_11_H_12_ClFN_7_ (M+H)^+^ 296.0827, found 296.0835. Mp: 108 ± 2 °C.

*6-Bromo-7-{2-[1-(2-fluoroethyl)-1H-*[1,2,3]*triazol-4-yl]-ethyl}-purine* (**8**). To a solution of **5a** (20 mg, 0.08 mmol) and FEA (9.2 mg, 0.10 mmol) in DMF (1.5 mL) was added sodium ascorbate (1.5 mg, 0.008 mmol) and CuSO_4_·5H_2_O (1.0 mg, 0.004 mmol) in H_2_O (0.1 mL) as described under the general procedure to give compound **8** in 37% yield (10 mg). ^1^H-NMR (CDCl_3_, 600 MHz): δ 8.82 (s, 1H, H-2), 8.08 (s, 1H, H-8), 7.27 (s, 1H, CH), 4.96 (t, *J*_1,2_ = 6.66 Hz, 2H, C*H_2_*CH_2_), 4.74 (dt, *J*_F,H_ = 46.81 Hz, *J*_1,2_ = 4.74 Hz, 2H, CH_2_F), 4.61 (dt, *J*_F,H_ = 27.13 Hz, *J*_1,2_ = 4.56 Hz, 2H, C*H*_2_CH_2_F), 3.35 (t, *J*_1,2_ = 6.60 Hz, 2H, CH_2_C*H_2_*). ^13^C-NMR (CDCl_3_): δ 161.3, 152.6, 150.0, 142.7, 133.1, 124.4, 123.3, 82.2, 81.0, 50.8, 50.6, 45.9, 28.1. Accurate mass (ESI-MS) *m/z* calcd for C_11_H_12_BrFN_7_ (M+H)^+^ 340.0322, found 340.0315. Mp: 120 ± 1 °C.

*6-Bromo-9-[1-(2-fluoroethyl)-1H-*[1,2,3]*triazol-4-ylmethyl]-purine* (**9**). To a solution of **4b** (80 mg, 0.34 mmol) and FEA (39 mg, 0.44 mmol) in DMF (3.5 mL) was added sodium ascorbate (5.9 mg, 0.034 mmol) and CuSO_4_·5H_2_O (4.2 mg, 0.017 mmol) in H_2_O (0.4 mL) as described under the general procedure to give compound **9** in 60% yield (41 mg). ^1^H-NMR (CDCl_3_, 600 MHz): δ 8.73 (s, 1H, H-2), 8.36 (s, 1H, H-8), 7.81 (s, 1H, CH), 5.59 (s, 2H, CH_2_), 4.78 (dt, *J*_F,H_ = 46.00 Hz, *J*_1,2_ = 4.20 Hz, 2H, CH_2_F), 4.67 (dt, *J*_F,H_ = 27.00 Hz, *J*_1,2_ = 4.80 Hz, 2H, C*H*_2_CH_2_F). ^13^C-NMR (CDCl_3_): δ 152.2, 150.3, 145.0, 143.5, 141.8, 134.3, 124.1, 82.0, 80.8, 51.0, 50.8, 39.1. Accurate mass (ESI-MS) *m/z* calcd for C_10_H_10_BrFN_7_ (M + H)^+^ 326.0165, found 326.0160. Mp: 103 ± 1 °C.

### 3.3. Radiochemistry

#### 3.3.1. One-pot Method for Cycloaddition of [^18^F]FEA to the Alkyne-Functionalized Purine Precursors

[^18^F]FEA was prepared as described by Glaser and Årstad. [2] Briefly, a mixture of Kryptofix 222 and potassium carbonate (30 mM : 15 mM dissolved in 0.5 mL acetonitrile-water = 4:1) was added to [^18^F]fluoride (~30 MBq) in water. The solvent was removed by heating at 90 °C under a stream of nitrogen. Acetonitrile (0.5 mL) was added, and the distillation continued. The procedure was repeated twice. After cooling to room temperature, a solution of 2-azidoethyl-4-toluenesulfonate (1.8 mg, 8.4 µmol in 0.4 mL anhydrous acetonitrile) was added. The reaction mixture was stirred at 80 °C. After 15 min, the crude mixture was allowed to reach ambient temperature prior to addition of the alkyne precursor (10 µmol in 0.15 mL acetonitrile), a solution of CuSO_4_·5H_2_O and bathophenanthrolinedisulfonic acid disodium salt hydrate (BPDS) (8 µmol each in 0.2 mL phosphate buffer 0.1 M, pH = 7.4), and sodium ascorbate (40 µmol in 0.1 mL H_2_O). After 1 min at room temperature the reaction was diluted with 1 mL H_2_O, and aliquots taken to determine the analytical and isolated RCYs.

*[^18^F]6-Chloro-7-[1-(2-fluoroethyl)-1H-*[1,2,3]*triazol-4-ylmethyl]-purine* ([^18^F]**6**). Hands-on reaction: analytical RCY: 60 ± 1% (n = 3); isolated RCY 41%. HPLC gradient system: the methanol content was increased from 5% to 12% over 7 min, then to 20% over 1 min and finally to 70% over 4 min. The flow rate was 3 mL/min and 5 mL/min for analytical and semi-preparative HPLC, respectively. Retention times of [^18^F]**6** were 5.6 min and 8.6 min for analytical and semi-preparative runs, respectively.

*[^18^F]6-Chloro-7-{2-[1-(2-fluoroethyl)-1H-*[1,2,3]*triazol-4-yl]-ethyl}-purine* ([^18^F]**7**). Hands-on reaction: analytical RCY: 76 ± 1% (n = 3). Automated synthesis: analytical RCY: 75 ± 1% (n = 3); isolated RCY was 40% when using a C-18 Plus cartridge. When using a C-18 light cartridge the RCY was 9 ± 2% (n = 3) after sterile filtration, with a specific activity of 5-7 GBq/µmol. Analytical HPLC was carried out with a Luna C18(2) column (3µm, 50 × 4.6 mm, Phenomenex) with a flow rate of 1 mL/min using the following gradient: the methanol content was increased from 5% to 70% in 13 min. Semi-preparative HPLC with a Chromolith® performance column and a flow rate of 5 mL/min was carried out using an isocratic solvent system consisting of 9% acetonitrile in water with 0.1% formic acid. Retention time of [^18^F]**7**: 7.1 min and 17 min for analytical and semi-preparative runs, respectively. The specific activity was measured using a Chromolith® performance RP18-e (100 × 4.6 mm) column with a flow rate of 5 mL/min [Figure 4(b)]. The methanol content was increased from 5% to 20% over 5 min, and then to 50% over 3 min. A calibration curve was obtained by correlating the area under the peak (UV = 265 nm) with the injected mass of the non-radioactive reference compound **7** across a predetermined range (measurements in duplicate, linear fit with R^2^ = 99.4%). Following formulation of [^18^F]**7**, an aliquot was analyzed and the correlation curve was used to estimate the total mass in the sample (UV integration from 2–8 min). The specific activity was calculated as the activity in the aliquot divided on the estimated mass expressed as µmol of **7**.

*[^18^F]6-Bromo-7-{2-[1-(2-fluoroethyl)-1H-*[1,2,3]*triazol-4-yl]-ethyl}-purine* ([^18^F]**8**). Hands-on reaction: analytical RCY: 55 ± 2% (n = 3); isolated RCY 55%. HPLC solvent gradient: the methanol gradient was increased from 5% to 20% in 5 min, kept constant at 20% for 2 min, and increased to 70% over 2 min. The flow rate was 3 mL/min and 5 mL/min for analytical and semi-preparative HPLC, respectively. Retention time of [^18^F]**8**: 5.6 min and 7.2 min for analytical and semi-preparative runs, respectively.

*[^18^F]6-Bromo-9-[1-(2-fluoroethyl)-1H-*[1,2,3]*triazol-4-ylmethyl]-purine* ([^18^F]**9**). Hands-on reaction: analytical RCY: 77 ± 1% (n = 3); isolated RCY 57%. Analytical HPLC was carried out with a Luna C18(2) column (3µm, 50 × 4.6 mm, Phenomenex) with a flow rate of 1 mL/min using the following gradient: the methanol content was increased from 20% to 45% in 6 min. Semi-preparative HPLC was carried out with a Chromolith® performance column using a flow rate of 5 mL/min. The methanol content was increased from 15% to 30% over 5 min. Retention time of [^18^F]**9**: 6.2 min and 4.1 min for analytical and semi-preparative runs, respectively.

## 4. Conclusions

Conditions for labeling of alkyne-functionalized 6-halopurines with [^18^F]fluoroethyl azide have been investigated. The use of the copper(II)/ascorbate catalytic system failed to yield the target triazoles in satisfactory yields, and addition of excess of copper(II) relative to the alkynes led to decomposition of the precursors. In contrast, when BPDS was used as an auxiliary copper(I) chelator the cycloaddition reaction proceeded almost immediately at room temperature to give the corresponding triazole-substituted purines. The high reaction efficiency in the presence of BPDS-copper(I) was exploited to develop a one-pot method for labeling that circumvents the need to distill [^18^F]fluoroethyl azide. The method enabled labeling of four 6-halopurines in 55–76% analytical radiochemical yield with short reaction time and under mild conditions (1 min at room temperature). The ability to carry out the reaction in one pot without the need for distillation makes this a highly convenient approach to prepare [^18^F]fluoroethyltriazoles.
